# The temporal dynamics of transition to psychosis in individuals at clinical high-risk (CHR-P) shows negative prognostic effects of baseline antipsychotic exposure: a meta-analysis

**DOI:** 10.1038/s41398-023-02405-6

**Published:** 2023-04-05

**Authors:** Andrea Raballo, Michele Poletti, Antonio Preti

**Affiliations:** 1 Chair of Psychiatry, Faculty of Biomedical Sciences, Università della Svizzera Italiana (USI), Lugano, Switzerland; 2 Cantonal Socio-psychiatric Organization (OSC), Public Health Division, Department of Health and Social Care, Repubblica e Cantone Ticino, Mendrisio, Switzerland; 3 Department of Mental Health and Pathological Addiction, Child and Adolescent Neuropsychiatry Service, Azienda USL-IRCCS di Reggio Emilia, Reggio Emilia, Italy; 4 Department of Neuroscience, University of Turin, Turin, Italy

**Keywords:** Prognostic markers, Clinical pharmacology

## Abstract

Meta-analytic evidence indicates that baseline exposure to antipsychotics (AP) in individuals at clinical high-risk for psychosis (CHR-P) is associated with an even higher risk of transition to psychosis. However, the temporal dynamics of such prognostic effect have not been clarified yet. This study was therefore designed to address this knowledge gap. We performed a systematic review and meta-analysis of all longitudinal studies published up to 31 December 2021 on CHR-P individuals identified according to a validated diagnostic procedure and reporting numeric data of transition to psychosis according to baseline antipsychotic exposure. 28 studies covering a total of 2405 CHR-P were included. 554 (23.0%) were exposed to AP at baseline, whereas 1851 (77.0%) were not. At follow-up (12 to 72 months), 182 individuals among AP-exposed (32.9%; 95% CI: 29.4% to 37.8%) and 382 among AP-naive CHR-P (20.6%; 18.8% to 22.8%) developed psychosis. Transition rates increased over time, with the best-fit for an ascending curve peaking at 24 months and reaching then a plateau, with a further increase at 48 months. Baseline AP-exposed CHR-P had higher transition risk at 12 months and then again at 36 and 48 months, with an overall higher risk of transition (fixed-effect model: risk ratio = 1.56 [95% CI: 1.32–1.85]; *z* = 5.32; *p* < 0.0001; Random-effect model: risk ratio = 1.56 [95% CI: 1.07–2.26]; *z* = 2.54; *p* = 0.0196). In conclusion, the temporal dynamics of transition to psychosis differ in AP-exposed vs. AP-naive CHR-P. Baseline AP exposure in CHR-P is associated with a persistently higher risk of transition at follow up, supporting the rationale for more stringent clinical monitoring in AP-exposed CHR-P. The insufficiency of more granular information in available primary literature (e.g., temporal and quantitative details of AP exposure as well as psychopathological dimensions in CHR-P) did not allow the testing of causal hypotheses on this negative prognostic association.

## Introduction

Research on clinical high-risk for psychosis (CHR-P) is a driving factor in the contemporary landscape of prevention-oriented youth mental health. In the last decades, the early detection field has been engaged in a robust effort to conceptualize and develop prognostic models for trans-diagnostic staging and individualized risk stratification [1, 2]. However, this exponentially increasing attention to operationalize predictors of psychosis has not been immune to inadvertent distortions in mainstream CHR-P literature, such as the lack of conceptualization of the potential prognostic role of baseline exposure to antipsychotics (AP) exposure as regards longitudinal outcome [3–5].

As revealed by a recent meta-analysis, the extension of such phenomenon is substantial: almost one out of five/four individuals enrolled as CHR-P in specialist centers are already undergoing AP treatment at the moment of the first evaluation, with intuitive consequences in terms of attenuation of the clinical presentation of CHR-P [6]. In this perspective, individuals already experiencing a First-Episode Psychosis might be surreptitiously considered as Attenuated Psychosis Syndrome due to the neglect of the pharmacological attenuation of symptoms [7]. Indeed, there is meta-analytic evidence that: a) baseline AP exposure in CHR-P individuals is associated with an even higher imminent risk of transition to psychosis in comparison with no-AP exposure (29% vs. 16%; risk ratio of transition 1.47) [8], an effect not due to a pre-test risk enrichment [9].

Considering clinical guidelines for the management of schizophrenia and psychotic disorders [10–12], the issue of baseline AP medication in CHR-P is important for optimal delivery of care as well as for improved risk stratification [8]. However, current evidences are insufficient to discriminate whether AP in CHR-P are overall protective, potentially detrimental or are rather to be considered a further risk flag [13, 14]. Furthermore, it is not clear whether the negative prognostic impact of baseline AP exposure is a transitory phenomenon, which progressively fades away during the follow up (i.e., baseline AP-exposure is a short-term index of increased imminent risk of transition) or whether it reveals a more enduring effect on the transition risk (indicating that baseline AP-exposure indexes a subgroup of CHR-P with persistently increased risk of imminent transition to psychosis).

On this background, this meta-analytical study was conceptualized to investigate the longitudinal effects of baseline AP exposure in CHR-P subjects in relation to the temporal dynamics of transition to psychosis, i.e., if the transition to psychosis in CHR-P subjects on AP at baseline follows distinct temporal trajectories as compared to CHR-P subjects who were not on AP at baseline.

## Materials and methods

### Data source and search strategy

This study has been set up according to the requirements of the Preferred Reporting Items for Systematic Reviews and Meta-Analyses (PRISMA) [15, 16]. The following search engines were probed: PubMed/Medline (https://pubmed.ncbi.nlm.nih.gov/) and the Cochrane Library (https://www.cochranelibrary.com/). The time interval was from inception up to 31 December 2021.

A combination of the following key terms was applied: “Ultra high risk” OR “Clinical high risk” OR “psychosis prodrome” OR “Psychosis Risk” AND “psychosis” AND “transition” OR “conversion”.

### Inclusion and exclusion criteria

The results of the search were inspected by two investigators with minimum 10 years of experience in the doing of systematic review and cross-checked by a third investigator expert in the field. The following criteria were applied to retain an article in the study: the article was written in English; it included samples with people diagnosed CHR-P based on a validated diagnostic procedure of assessment (i.e. a validated diagnostic interview); it reported numeric data about the sample and the outcome at a predefined follow-up time; it reported data on psychometric transition to psychosis (i.e., on the same diagnostic instrument used for CHR-P ascertainment), as defined according to pre-specified criteria, as one of the outcomes; it detailed raw data on AP baseline exposure in relation to the transition outcome (i.e., specified for both subjects transitioned to psychosis and subjects not transitioned to psychosis how many were or were not exposed to AP at baseline).

### Data extraction and quality assessment

Independent studies were included when they matched the inclusion criteria. Articles that were unrelated to the main topic (e.g., studies on genetic markers) and duplicates (e.g., articles repeatedly reporting the results of the same trial with overlapping samples) were excluded. In case of overlapping samples, we included the sample reporting data on AP data exposure in relation to the transition outcome; in case of duplicates with similar data we included those with the larger sample.

**Fig. 1:**
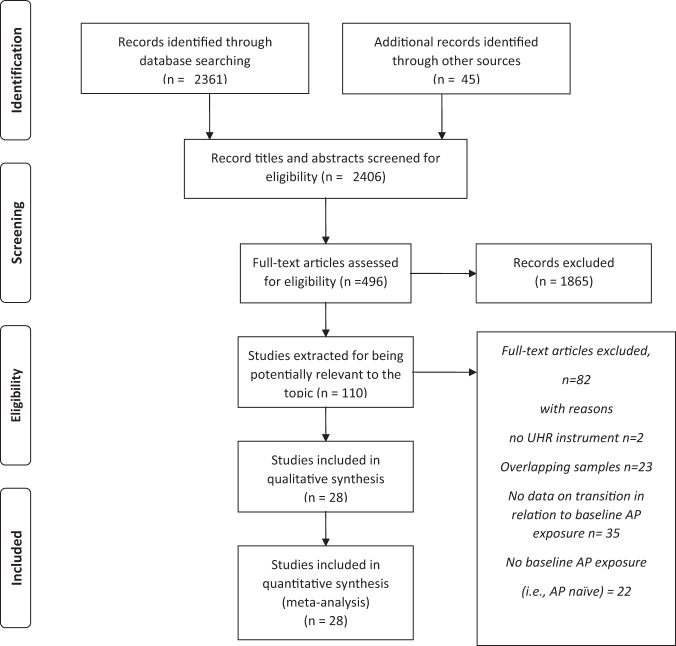
PRISMA flowchart. Flowchart of study selection.

When discrepancies did arise in judging about correspondence of the article to the inclusion criteria or about considering it a duplicate publication, a discussion with a third experienced researcher was used to take a final decision. All references of the scanned articles and of the available reviews on the topic were looked for potentially missed studies. At the end of this procedure, 28 independent studies were included in the systematic analysis and the subsequent meta-analysis (Fig. 1: PRISMA flowchart).

Two experienced investigator extracted the following variables on the basis of a predefined protocol: authors and year of publication of the study; location of the study; criteria and instrument for diagnosis; criteria for transition to psychosis; sample size at baseline and at follow-up; mean age in the sample at baseline; gender ratio in the sample; data on AP exposure (yes/no) on the basis of outcome(transition/no transition); duration of the follow-up; number of cases that transitioned psychosis at the end of follow-up by group. Quality assessment was rated according to the Newcastle-Ottawa Scale (NOS) [17]. The scale assigns an asterisk when the criterion is meet, corresponding to a low risk of bias (Table S1 in supplementary material). Otherwise, a high risk of bias was assigned. The overall quality of included studies was categorized, based on the adherence to the expected qualitative topics, as “good” or at low risk of bias, “fair” or with some concerns for bias, or “poor” or at high risk of bias. Again, when discrepancies did arise in applying the quality assessment tool, a discussion within the research team was used to take a final decision. The graphic presentation of the risk-of-bias assessment summary plot was created with the “robvis” package running in R [18].

### Statistical analysis

Preliminary analysis was done with the IBM SPSS Statistics for Windows, Version 26.0. Subsequent analysis was done with the “meta” package [19] and the “metafor” package [20] running in R version 4.0.2 [21]. Threshold for statistically significant results was set at p < 0.05, with both interval of 95% confidence interval (CI) above or below the unit (depending on the direction of the effect).

Transition risk was calculated as the proportion of transition to psychosis. The variance-stabilizing Freeman and Tukey [22] double arcsine transformation was used to estimate all proportions. Between- and within-studies variance were estimated with the τ2 statistics using Empirical Bayes estimator [23] and its 95% CI, which was calculated by using the Q-Profile method [20] with Knapp and Hartung correction [24]. Continuity correction of 0.5 was applied in studies with zero cell frequencies.

Estimates of transition risk were reported by type of exposure to AP at baseline (yes = AP+; no = AP–) and by time to follow-up (in the interval from 12 to 72 months that depends on the time frame of the source primary study). The results of the fixed effects model were given preference since the number of studies and sample size by time and AP exposure were too few and too small to allow any extrapolation of the estimates from the included studies to the population from which they are extracted (as expected by the random-effects model [20]). Heterogeneity was assessed with Cochran‟s *Q* and I2 statistics [25]. A low *p*-value (i.e., *p* < 0.10) of the Q-statistic indicates that variation in the study-specific effect estimates is due to heterogeneity beyond that depending on sampling error. The higher the I2, the greater the impact of the variance in true effects beyond that depending on sampling error [26]. Publication bias was tested by funnel plot, for evidence of asymmetry, and the Egger [27] and Begg test [28]. Since there were less than ten studies per group, no funnel plot or result of the Egger‟s or Begg‟s test was reported for subgroup analysis. Meta-regression was applied to estimate the impact of gender (proportion of women in the sample), age, follow-up, and dropout on transition risk by exposure to APs.

The temporal dynamics of transition to psychosis was investigated as a cumulative transition curve. To this aim, the baseline samples of each study were summed by creating a single population of CHR-P patients at risk of transition, and their combined transition was calculated by summing the number of transitions from each study at each follow-up and dividing by the global number of patients at baseline. Fitting of the curve was tested by linear and polynomial (quadratic and cubic) regression. The models were compared by adjusted *R*^2^ and ANOVA, to test whether the more complex model was significantly better at capturing the data than the simpler model. The “ggplot2” [29] and the “ggrepel” [30] packages running in R were used to graph the curves.

### Search results

Overall, 28 studies [31–58] were included in this meta-analysis (see Table 1). These studies included 554 participants that were exposed to AP at baseline and 1851 that were not.

**Table 1:** Studies included in the meta-analysis and reporting raw baseline data on AP exposure in relation to transition to psychosis.

Study	Site	Baseline CHR sample	Follow up	Follow up sample	Raw transitions	UHR instrument	Baseline AP exposure	Mean age (SD)	Gender (F)	Conv. on AP baseline	Conv. no AP baseline	Nonconv. on AP baseline	Nonconv. no AP baseline
		*n*	Months	*n*	*n*		%	Years	%	*n*	*n*	*n*	*n*
Borgwardt et al. (2007)	Switzerland	35	12	35	12	BSIP	9	23,7 (5,6)	37	1	11	2	21
van Tricht et al. (2010)	Netherlands	61	36	61	18	SIPS	26.2	19.6 (2.7)	31.1	7	11	9	34
Walker et al. (2010)	USA	56	60	56	14	SIPS	16,6	14,2 (1,6)	33,3	1	13	8	34
Bearden et al. (2011)	USA	59	24	54	21	SIPS	19.5	17.1 (3.8)	29.6	4	17	6	27
Liu et al. (2011)	Taiwan	59	36	59	21	SIPS	79.6	21.5 (4.0)	44.1	20	1	27	11
Ziermans et al. (2011)	Netherlands	72	12	58	9	SIPS	24.1	15.3 (1.0)	38.9	1	8	13	36
Schossler et al. (2012)	USA	125	12	84	27	SIPS	22.4	16.9 (3.5)	38	13	14	15	42
De Vylder et al. (2014)	USA	100	30	100	26	SIPS	14	20.1 (3.8)	24	4	22	10	64
Katsura et al. (2014)	Japan	106	30	82	14	CAARMS	37.3	20 (4.3)	62.3	3	11	31	37
Perez et al. (2014)	USA	38	24	31	15	SIPS	28.9	17.4 (3.5)	39.5	5	10	3	13
Schultze-Lutter et al. (2014)	Germany	194	24	194	74	SIPS	13.8	24.9 (6.0)	37	14	67	14	99
Bedi et al. (2015)	USA	34	30	34	5	SIPS	20.8	21.4 (3.5)	67.6	1	4	6	23
Katagiri et al. (2015)	Japan	41	12	41	7	SIPS	17.1	23.1 (6.7)	75.6	7	0	0	34
Labad et al. (2015)	Spain	39	12	39	10	PANSS	17.9	22.3 (4.6)	30.8	4	6	3	26
Brucato et al. (2017)	USA	200	24	200	60	SIPS	5.5	20.0 (3.9)	27	9	51	22	118
Kotlicka-Antczak et al. (2017)	Poland	81	62	65	19	CAARMS	18,5	18,7 (3,5)	51,9	7	12	4	42
Collin et al. (2018)	China	158	13	158	23	SIPS	15.2	18.8 (4.9)	49.4	6	17	18	117
Bang et al. (2019)	Korea	77	24	77	16	SIPS	31.2	19.9 (3.4)	40.3	4	12	20	41
Hamilton et al. (2019)	USA	43	28	43	15	SIPS	18,6	16,9 (3,5)	37,2	4	11	1	17
Zarogianni et al. (2019)	Switzerland	37	48	35	16	BSIP	20	25.3 (6.3)	40	6	10	1	18
Bourgin et al. (2020)	France	46	22	27	11	CAARMS	40,7	17,6 (3,7)	14,8	5	6	6	10
Demars et al. (2020)	France	92	12	92	36	CAARMS	59.8	21.1 (3.2)	38.0	22	14	34	22
Modinos et al. (2020)	UK	76	72	76	13	CAARMS	12	22.5 (3.6)	44.7	1	12	8	55
Nagele et al. (2020)	Germany	24	15	24	8	SIPS	33.3	21.3 (3.5)	50	3	5	5	11
Yoviene-Sikes et al. (2020)	USA	764	12	431	33	SIPS	20.4	19.1 (4.4)	41.8	21	12	67	331
Grent’t-Jong et al. (2021)	Scotland	116	12	110	13	CAARMS	2	22 (4,5)	70,7	0	13	2	95
Kristensen et al. 2021	Denmark	110	12	110	10	CAARMS	33,6	24 (4)	52,7	4	6	33	67
Tateno et al. (2021)	Japan	39	24	39	11	CAARMS	23.1	18.5 (4.2)	35.9	5	6	4	24

### Study characteristics and qualitative synthesis

Overall, 9 studies included participants from United States, 5 from Asian Countries (2 Japan, 1 China, 1 South Korea, 1 Taiwan) and 14 from Europe (3 Germany, 2 France, 2 Netherlands, 2 Switzerland, 2 UK, 1 Denmark, 1 Poland, 1 Spain). All studies included details about age and gender ratio. Studies do vary hugely as far as sample size, time to follow-up, age and gender ratio were concerned.

Mean age at baseline in the 28 studies was 20.2 ± 2.8, ranging from 14.2 to 25.3 years old. Proportion of women was 42% on average, ranging from 15% to 75%. 2 studies presented a sample including exclusively children or adolescents, 4 studies with only adult participants (aged 18 years old and older) and 22 studies based on mixed samples, with both children/adolescents and adults.

Sample size at baseline ranged from 24 to 764, with average sample size = 100. Sample size at follow up ranged 24 to 431, being on average = 84. Dropout at the end of the follow-up was rare, occurring in 10 studies out of 28, with dropouts ranging from 5.2% to 43.6%; however, just four studies had more than 20% of dropouts.

Time to follow-up was up to 12 months in 11 studies, around 24 months in 7 studies, and 30 months or longer in 10 studies.

As far as the tool for the diagnosis, there were 8 studies using the Comprehensive Assessment of At Risk Mental States (CAARMS) [59], 1 study using the Positive and Negative Syndrome Scale (PANSS) [60], 2 study using the Basel Screening Instrument for Psychosis [61] and 17 studies using the Structured Interview for Prodromal Syndromes (SIPS) [62]. Except one, almost all studies included help-seeking subjects.

Quality was poor in 5 studies and good in the other 23 (see Table S1). The proportion of participants with exposure to AP at baseline was substantial, ranging from 2% up to 79.6% pending on the study.

The participants who were already exposed to AP at baseline (from herein upon, “cases”) were 554 (23.0% of the whole sample; range: 2 to 88; average per sample: 19), while those without exposure to AP at baseline (“controls”) were 1851 (77.0%; range: 12 to 343; average per sample: 64). At the end of the period of observation, i.e., the follow-up as reported in the study, 182 (32.9%; 95% CI: 29.0% to 36.9%) participants developed psychosis among the cases against 382 among the controls (20.6%; 18.9% to 22.6%).

The overall quality of the studies was reasonably good (Fig. A1 in the supplementary material). Some bias did occur in the comparability of the groups and in the length of follow-up. A high risk of bias was rated for less than 20% of the studies.

### Meta-analysis of transition risk by time to follow-up and exposure to antipsychotics

**Fig. 2:**
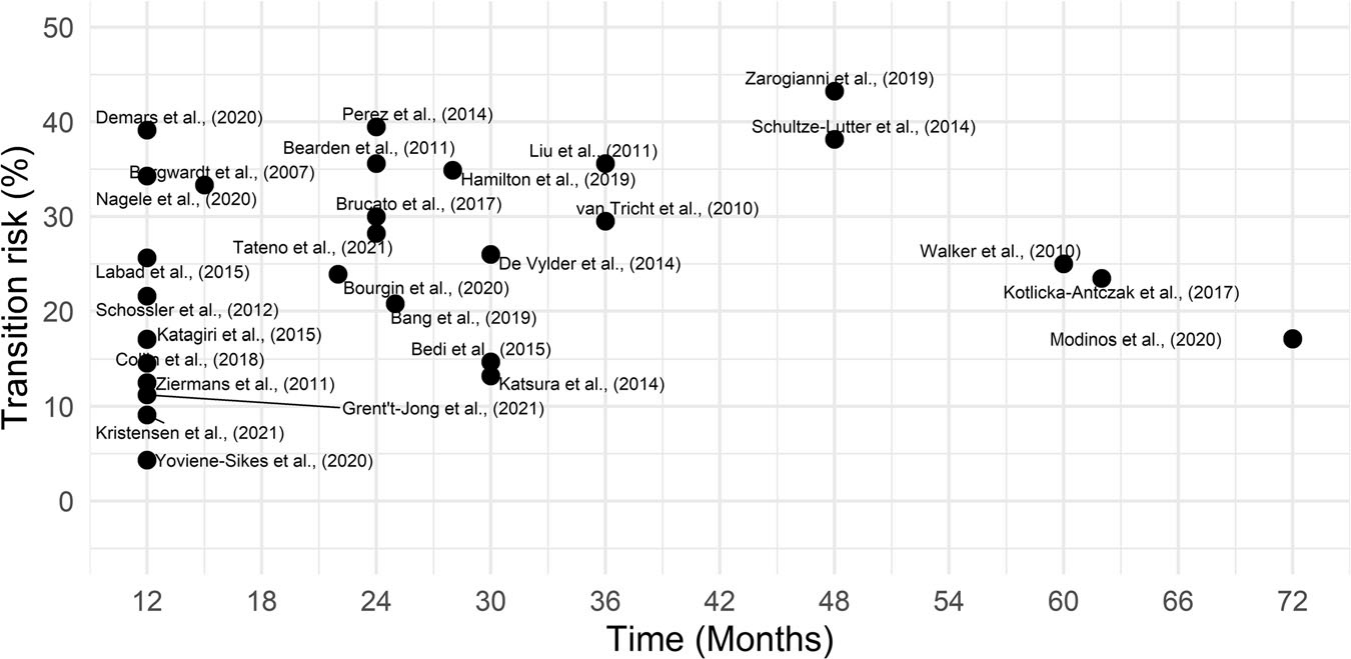
Distribution of transition risk by study and follow-up.

In the included studies, overall transition rates tended to increase over time (Fig. 2).

The best-fit is for an ascending curve peaking at 24 months and reaching then a plateau, with a further increase at 48 months and thereafter a decline. The adjusted *R*^2^ for the linear, quadratic and cubic regression were, respectively, 0.877, 0.995, and 0.993; the quadratic model was superior to the linear (ANOVA *F* = 117.32; *p* = 0.0004) and equivalent to the cubic model (ANOVA *F* = 0.015; *p* = 0.911).

When the dataset was split into the subsample with baseline exposure to AP (AP+) and those who did not received AP at baseline (AP–), we found that AP + had a higher transition risk at 12 months and then again at 36 and 48 months (Table 2). Heterogeneity was substantial at 12 months and declined thereafter, with a new peak at 48 months and over.

**Table 2:** Distribution of transition risk by time to follow-up and exposure to antipsychotics, with risk ratio.

Follow-up (months)	Exposure	*k*	*n*	Transition risk	95% CI	*Q*	*p*-value	Tau^2^	*I*^2^
12	AP+	11	274	27.4%	21.6%–33.6%	42.1	<0.001	0.063	76%
	AP–	11	908	9.5%	7.5%–11.6%	83.9	<0.001	0.035	88%
				RR = 2.10	1.64–2.69	31.1	<0.001	0.651	68%
24	AP+	8	98	35.4%	25.5%–45.9%	12.6	0.05	0.019	52%
	AP–	8	363	30.7%	25.9%–35.7%	7.5	0.28	0.002	20%
				RR = 1.20	0.90–1.61	7.0	0.43	0.0008	1%
30	AP+	3	55	12.8%	4.3%–24.0%	2.8	0.25	0.005	28%
	AP–	3	161	22.8%	16.5%–29.7%	1.2	0.54	0.0	0%
				RR = 0.71	0.36–1.38	2.0	0.37	0.0	0%
36	AP+	2	63	42.8%	30.5%–55.5%	0.1	0.92	0.0	0%
	AP–	2	57	20.2%	10.2%–32.2%	1.3	0.26	0.004	23%
				RR = 2.51	1.16–5.43	1.0	0.32	0.002	0%
48	AP+	2	35	57.7%	40.1%–74.5%	2.8	0.09	0.038	64%
	AP–	2	194	39.6%	32.7%–46.7%	0.2	0.67	0.0	0%
				RR = 1.44	1.02–2.03	3.3	0.07	0.152	70%
60	AP+	2	20	38.2%	17.1%–61.5%	5.6	0.02	0.111	82%
	AP–	2	101	24.7%	16.7%–33.7%	0.4	0.53	0.0	0%
				RR = 1.62	0.84–3.10	3.6	0.06	1.398	72%
72	AP+	1	9	11.1%	0.0%–41.8%	--	--	--	--
	AP–	1	67	17.9%	9.6%–29.2%	--	--	--	--
				RR = 0.62	0.09–4.22	--	--	--	--

Fixed-effects model.

**Fig. 3:**
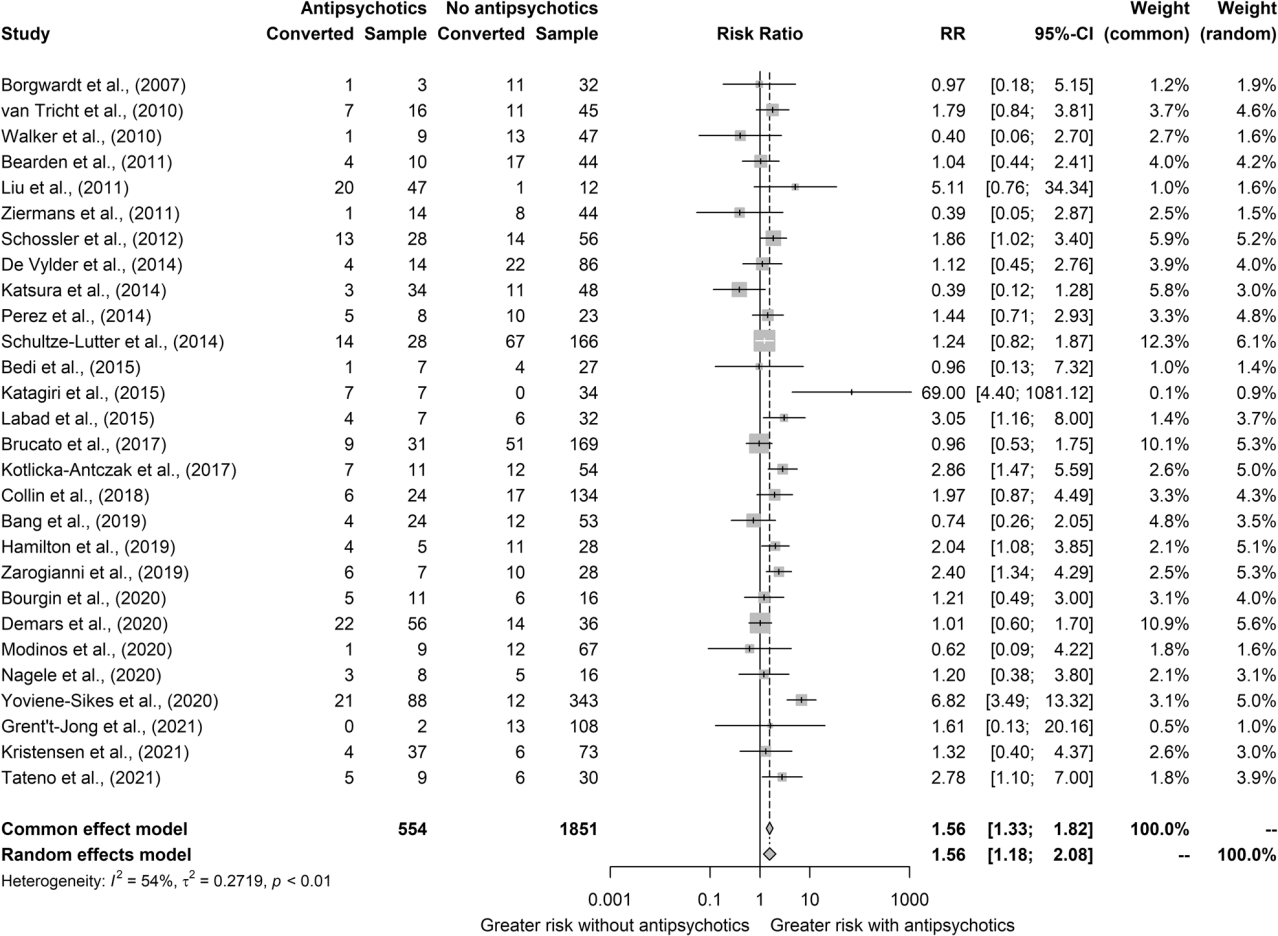
Forest plot of the risk ratio of conversion to psychosis between CHR who were or were not exposed to antipsychotics at baseline.

Overall, AP+ participants had a higher risk of transition than AP- (Fixed-effect model: risk ratio = 1.56 [95% CI: 1.33–1.82]; *z* = 5.61; *p* < 0.0001; Random-effect model: risk ratio = 1.56 [1.18–2.08]; *z* = 3.22; *p* = 0.0033) (Fig. 3). Heterogeneity was moderate to substantial (Cochran’s *Q* = 58.3; *df* = 27; *p* < 0.001; *I*^2^ = 54% [29%−70%]). There was a modest asymmetry at the funnel plot, because of an influential sample [43], but no evidence of publication bias at the Egger’s or Begg’s test (Fig. A2 in the supplementary material).

Gender (estimate = 0.011; s.e. = 0.012; *t* = 0.849; df = 26; *p* = 0.403; amount of heterogeneity accounted for = 0.0%), age (estimate = 0.026; s.e. = 0.052; *t* = 0.507; df = 26; *p* = 0.617; amount of heterogeneity accounted for = 0.0%), duration of follow-up (estimate = −0.005; s.e. = 0.009; *t* = −0.581; df = 26; *p* = 0.566; amount of heterogeneity accounted for = 0.0%), and dropout (estimate = 0.011; s.e. = 0.010; *t* = 1.153; df = 26; *p* = 0.259; amount of heterogeneity accounted for = 2.8%) did not influence risk ratio in transition to psychosis in the meta-analyzed studies. Tools used to ascertain the CHR-P status and the transition to psychosis, too, did not impact on the risk ratio (F[3;24] = 0.419, *p* = 0.741), and in particular, studies using SIPS (*n*. = 17; RR = 1.57 [95% CI: 1.04–2.38]; *I*^2^ = 60.3%) had not a statistically higher RR than studies using other diagnostic tools (*n*. = 11; RR = 1.54 [0.99–2.39]; *I*^2^ = 44.4%).

Quality of the studies, instead, had a relationship with the chance that patients exposed to AP at baseline had a transition to psychosis at follow-up: studies of high quality, hence a low risk of bias, reported higher risk of transition to psychosis in patients exposed to AP at baseline (*n*. = 23; RR = 1.68 [1.77–2.41]; *I*^2^ = 56.8%) than studies of low quality, hence at high risk of bias (*n*. = 5; RR = 1.14 [1.01–1.30]; *I*^2^ = 0.0%); between groups difference: *Q* = 4.72; df = 1; *p* = 0.030.

We repeated the analysis by excluding samples with more than 20% dropouts at the end of follow-up. In the remaining 24 studies with no or less than 20% dropouts, still AP+ participants had a higher risk of transition than AP– (Fixed-effect model: risk ratio = 1.43 [95% CI: 1.20–1.70]; *z* = 4.04; *p* < 0.0001; Random-effect model: risk ratio = 1.51 [95% CI: 1.17–1.94]; *z* = 3.36; *p* = 0.0027) (Fig. A3 in supplementary material). Heterogeneity was greatly reduced by the exclusion of the four studies with more than 20% dropouts at follow-up: Cochran’s *Q* = 33.7; df = 24; *p* = 0.089; *I*^2^ = 29% [0.0% − 56%]).

### Cumulative transition curves

**Fig. 4:**
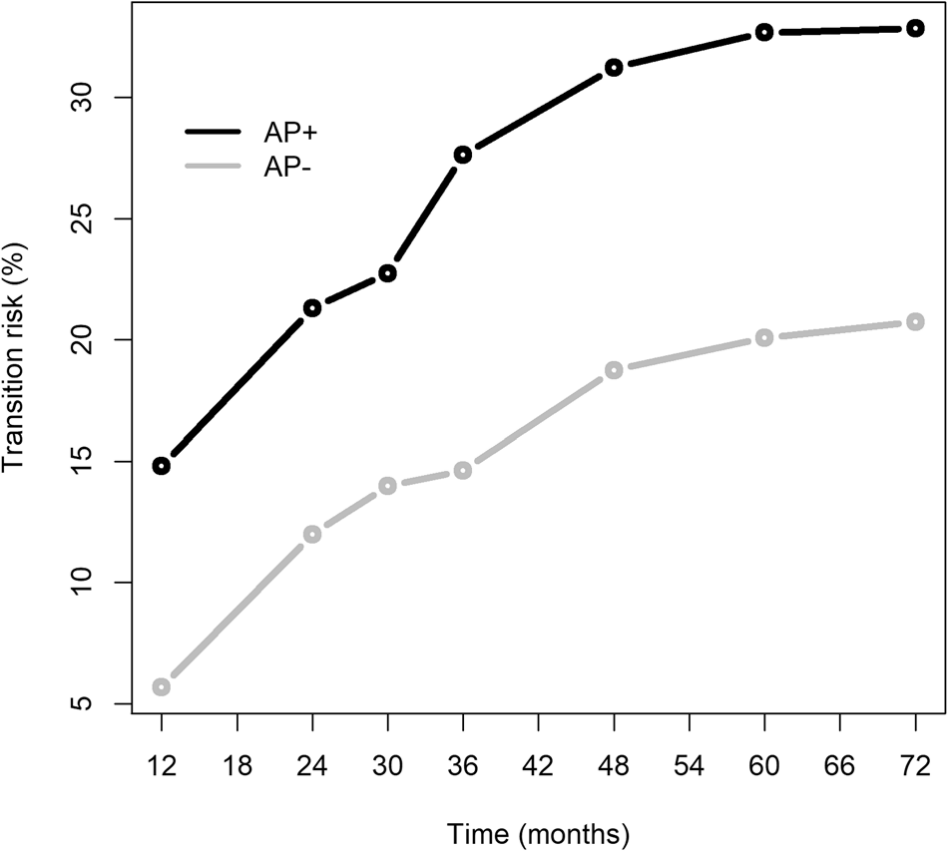
Cumulative transition curves in CHR patients who were exposed (AP+) or not exposed (AP–) to antipsychotics at baseline.

The temporal dynamics of cumulative transition to psychosis confirmed the divarication of risk trajectories between AP+ and AP– participants. Cumulative transition rates were higher in CHR-P subjects exposed to APs at baseline than in those not exposed to APs at baseline at any point in time (Fig. 4). This depended primarily on a higher fraction of transition to psychosis in APs-exposed participants at 12 months: 27.4% vs. 9.5% (Table 2 for details). Essentially, CHR-P subjects exposed to APs at baseline were quicker to transition to psychosis than unexposed subjects.

The temporal dynamics of cumulative transition to psychosis in the AP+ group showed a linear increase, with adjusted *R*^2^ = 0.863, with an advantage of the quadratic curve (adjusted *R*^2^ = 0.979; ANOVA *F* = 28.99; *p* = 0.006) but no further increment at the cubic model (adjusted *R*^2^ = 0.979; ANOVA *F* = 1.02; *p* = 0.386).

The temporal dynamics of cumulative transition to psychosis in the AP- group was comparable, with an increase leaning towards a plateau at 24 to 36 months (Fig. A4 in the supplementary material).

In this group, the adjusted *R*^2^ for the linear, quadratic and cubic regression were, respectively, 0.880, 0.986, and 0.983, and the polynomial (quadratic) curve had a more neat best-fit in comparison to the linear regression (ANOVA *F* = 39.83; *p* = 0.003) but did not differ from the cubic curve (ANOVA *F* = 0.21; *p* = 0.681).

## Discussion

The study (1) confirms the negative prognostic effect of baseline exposure to AP in help-seeking youth accessing early intervention centers with symptoms compatible with the CHR-P status [8] and (2) reveals the enduring nature of such effect along the follow-up to 48 months. The deconstruction of the temporal dynamics of cumulative transition to psychosis indicates that CHR-P individuals exposed to AP at baseline tend to have an immediate increase in the imminent risk, with a trend towards persistent boost across time. Those not exposed to AP at baseline reach a plateau between 24 and 36 months, albeit with a similar temporal dynamics to those exposed to AP at baseline.

Such effect of AP exposure might have different explanations:

(1) pro-psychotic (i.e., iatrogenic) effect of AP in CHR-P via dopamine-sensitization

(2) temporary AP masking effect: i.e., AP may attenuate the clinical presentation of the first psychotic episode in terms of apparent CHR-P status, but medicated individuals maintain a higher proclivity to later trespass the psychometric threshold for psychosis at follow-up;

(3) delaying effect of AP on the transition to psychosis: i.e., APs are prescribed to individuals with increasing clinical severity although still formally within the psychometric criteria for CHR-P at the moment of prescription; APs delay the transition to psychosis but are insufficient to prevent it because after a while the severity of the condition overcomes the therapeutic effects of the drug.

### Hypothesis 1: Pro-psychotic effect of AP in CHR-P via sensitization

APs may exert per se a psychotogenic action in CHR-P subjects, via a sensitization effect on the dopaminergic neurons [63]. Indeed, the persistent block of the pre- and post-synaptic receptors might cause a hypersensitivity to dopaminergic discharge [64–66]. AP-induced hypersensitivity to dopamine has been related to rebound psychosis after discontinuation of treatment, withdrawal discontinuation syndromes, and tardive dyskinesia [67]. Eventually, supersensitivity to dopamine induced by exposure to AP, and the consequent drug-induced upregulation of D2 function, may result in tolerance to their therapeutic effects. Although AP-induced hypersensitivity to dopamine has been reported after long-term treatment in humans, in animals, tolerance to the behavioral effects of AP may be established in a few days [68]. Moreover, some evidence suggests that dopamine super-sensitization may only take 3 months to develop in patients at their first treatment with AP [64, 69]. Drug discontinuation or dose reduction after the establishment of the targeted effect, as well as dopamine perturbation because of psychological stress and substance use [69], may precipitate transition to psychosis in APs-exposed CHR-P patients enrolled in early intervention services. From the perspective of prevention of dopamine super-sensitization, the use of AP with minimal or no movement disorders or partial agonists (e.g., aripiprazole) has been suggested [68, 69], although there is no clear evidence in so far that these strategies are really effective [69].

### Hypothesis 2: Masking effect of AP “attenuating” the clinical presentation of psychosis

Individuals exposed to AP may have already transitioned towards psychosis, yet the ongoing AP treatment might have prevented the psychometric identification of these “pharmacologically attenuated first-episode psychosis” cases [7]. Crucially, this group is not anymore in a high-risk condition but rather it has already reached the endpoint outcome (i.e., the first-episode psychosis) although unrecognized. Nonetheless, individuals in “pharmacologically attenuated first-episode psychosis” might present a higher proclivity to undergo a “psychometric” transition to psychosis at follow-up (and subsequently considered as transited to FEP), due to insufficient stabilization of positive psychotic symptoms.

### Hypothesis 3: Delaying effect of AP “postponing” transition to psychosis

CHR-P individuals already on AP at baseline enrollment probably present more severe ongoing psychopathology and probably more rapidly worsening symptoms, globally depicting a whole clinical severity inducing the treating staff to prescribe AP before the clear emergence of full-blown positive symptoms. Although these CHR-P individuals might have an accelerated progression towards psychosis, AP may temporarily delay the actual transition to psychosis. Therefore, they correctly fulfill baseline CHR-P criteria, yet they develop a full psychotic state rather soon afterwards. Thus, this specific group is a subpopulation with the highest imminent risk of transition within CHR-P individuals (i.e., a Hyper-CHR-P group) and, conversely, baseline AP prescription is better regarded as a red warning flag for enhanced risk of transition to psychosis. Findings of two randomized controlled trials were in favor of the latter hypothesis (Delaying effect of AP): these studies tested the preventive action of low doses AP against placebo in CHR-P help-seekers [70, 71] and found low-doses AP to be able to reduce transition to psychosis on the short term (6 months) when compared to placebo, but the protective effects fade down at twelve months [71, 72].

Distinguishing pharmacologically attenuated first-episode psychosis cases (hypothesis 2) from CHR-P subjects with faster progression towards psychosis (hypothesis 3) is an essential step forward in the field, particularly for the issue of risk stratification, prognostic precision and treatment optimization. In this respect, the concept of time-dependent trajectories adopted in the current meta-analysis study may be a key feature to operate such distinction. Indeed, CHR-P individuals with rapid progression towards psychosis are likely to be recognized relatively early as transitioned cases, whereas “pharmacologically attenuated first-episode psychosis” cases are presumably more likely to persist into an “attenuated symptom condition” until additional factors (social stress, substance use, and/or discontinuation of the AP treatment) intervene. Overall, as revealed by the clearly separated temporal dynamics of the transition curves (Fig. 4), baseline AP-exposed CHR-P individuals reveal a relatively persistent higher risk of transition to psychosis already at 12 months follow up, whereas AP-naive CHR-P individuals progressively increase their risk up to 24 months and then tend to reach a risk plateau, which is anyhow substantially lower than AP-exposed CHR-P at any time point during the follow up.

### Limitations and strengths of the study

The current meta-analysis cannot overcome the intrinsic limits of the available literature in the field, particularly with respect to suboptimal transparency in reporting baseline (as well as intercurrent) medication exposure in available samples [5, 8, 13]. Moreover, available information did not specify why AP were prescribed at baseline, whether AP-exposed cases have continued to receive APs until conversion to psychosis or whether the prescription was discontinued because of other clinical reasons (e.g., side-effects). Similarly, dosage and concomitant treatment with other psychotropic drugs (e.g., antidepressants, anxiolytics, mood stabilizers) was only inconsistently reported. We also lacked details about the baseline symptom profile or dropouts at follow-up in relation to drug prescription. This prevented more detailed analyses (e.g., imputation or the best-worst case scenario for dropouts), Nonetheless, introducing a temporal angle to the analysis of transition (i.e., looking at the temporal dynamics of transition to psychosis) has never been attempted before and enriches the resolution of the current results, revealing the persistent nature of transition risk enhancement associated with baseline AP exposure, which extends up to 48 months.

## Conclusions

Despite its intuitive potential prognostic impact, baseline AP exposure in individuals at CHR-P has received surprisingly marginal attention until recently. The current study confirms the negative prognostic effect of such exposure (which is associated with higher imminent risk of transition to psychosis in CHR-P individuals) and indicates that the temporal dynamics of transition in AP-exposed vs. AP-non exposed CHR-P is substantially different. Specifically, AP-exposed CHR-P individuals show a persistently elevated risk of transition to psychosis up to 48 months after the initial assessment and represent a “Hyper-CHR-P” subgroup, which presumably requires more intensive monitoring and treatment support.

## Supplementary information

Supplementary materials

Table S1

Figure S1

Figure S2

Figure S3

Figure S4

## References

[1] Rosen M, Betz LT, Schultze-Lutter F, Chisholm K, Haidl TK, Kambeitz-Ilankovic L (2021). Towards clinical application of prediction models for transition to psychosis: a systematic review and external validation study in the PRONIA sample. Neurosci Biobehav Rev.

[2] Sanfelici R, Dwyer DB, Antonucci LA, Koutsouleris N (2020). Individualized diagnostic and prognostic models for patients with psychosis risk syndromes: a meta-analytic view on the state of the art. Biol Psychiatry.

[3] Raballo A, Poletti M, Carpenter W (2019). Rethinking the psychosis threshold in clinical high risk. Schizophr Bull.

[4] Raballo A, Poletti M (2022). Overlooking the transition elephant in the Ultra-High-Risk room: are we missing functional equivalents of transition to psychosis?. Psychol Med.

[5] Raballo A, Poletti M, Preti A (2021). Individualized diagnostic and prognostic models for psychosis risk syndromes: do not underestimate antipsychotic exposure. Biol Psychiatry.

[6] Salazar de Pablo S, Catalan A, Fusar-Poli P (2020). Clinical validity of DSM-5 Attenuated Psychosis Syndrome. Advances in diagnosis, prognosis and treatment. JAMA Psychiatry.

[7] Raballo A, Poletti M, Preti A (2020). Attenuated psychosis syndrome of “pharmacologically attenuated first episode psychosis? An undesirably widespread confounder. JAMA Psychiatry.

[8] Raballo A, Poletti M, Preti A (2020). Meta-analyzing the prevalence and prognostic effect of antipsychotic exposure in clinical high-risk (CHR): when things are not what they seem. Psychol Med.

[9] Raballo A, Poletti M, Preti A (2021). Negative prognostic effect of baseline antipsychotic exposure in Clinical High Risk for psychosis (CHR-P): is pre-test risk enrichment the hidden culprit?. Int J Neuropsychopharmacol.

[10] Galletly C, Castle D, Dark F, Humberstone V, Jablensky A, Killackey E (2016). Royal Australian and New Zealand College of Psychiatrists clinical practice guidelines for the management of schizophrenia and related disorders. Aust N Z J Psychiatry.

[11] Schmidt SJ, Schultze-Lutter F, Schimmelmann BG, Maric NP, Salokangas RK, Riecher- Rössler A (2015). EPA guidance on the early intervention in clinical high risk states of psychoses. Eur Psychiatry.

[12] National Institute for Health and Care Excellence. Psychosis and schizophrenia in adults: prevention and management. No. 178, NICE Clinical guideline; 2014. 32207892

[13] Raballo A, Poletti M, Preti A (2021). Antipsychotic treatment in clinical high risk for psychosis: protective, iatrogenic of further risk flag?. Aust N Z J Psychiatry.

[14] Zhang T, Xu L, Tang X, Wei Y, Hu Q, Hu Y (2020). Real-world effectiveness of antipsychotic treatment in psychosis prevention in a 3-year cohort of 517 individuals at clinical high risk from the SHARP (ShangHai At Risk for Psychosis). Aust N Z J Psychiatry.

[15] Moher D, Liberati A, Tetzlaff J, Altman DG (2009). Preferred reporting items for systematic reviews and meta-analyses: the PRISMA statement. BMJ.

[16] Page MJ, McKenzie JE, Bossuyt PM, Boutron I, Hoffmann TC, Mulrow CD (2021). The PRISMA 2020 statement: An updated guideline for reporting systematic reviews. J Clin Epidemiol.

[17] Wells GA, Wells G, Shea B, Shea B, O’Connell D, Peterson J, et al. The Newcastle-Ottawa Scale (NOS) for assessing the quality of nonrandomised studies in meta-analyses, 2014. http://www.ohri.ca/programs/clinical_epidemiology/oxford.asp

[18] McGuinness LA. Robvis: An R package and web application for visualising risk-of-bias assessments. 2019. Available at https://github.com/mcguinlu/robvis 10.1002/jrsm.1411 32336025

[19] Schwarzer G, Carpenter JR, Rücker G. Meta-analysis with R. Springer; 2015.

[20] Viechtbauer W (2010). Conducting meta-analyses in R with the metaphor package. J Stat Softw.

[21] R Core Team. R: A language and environment for statistical computing. Vienna, Austria: R Foundation for Statistical Computing; 2020. URL. https://www.R-project.org/

[22] Freeman MF, Tukey JW (1950). Transformations related to the angular and the square root. Ann Math Stat.

[23] Veroniki AA, Jackson D, Viechtbauer W, Bender R, Bowden J, Knapp G (2016). Methods to estimate the between-study variance and its uncertainty in meta-analysis. Res Synth Methods.

[24] Knapp G, Hartung J (2003). Improved tests for a random effects meta-regression with a single covariate. Stat Med.

[25] Huedo-Medina TB, Sánchez-Meca J, Marín-Martínez F, Botella J (2006). Assessing heterogeneity in meta-analysis: Q statistic or I2 index?. Psychol Methods.

[26] Borenstein M (2020). Research Note: In a meta-analysis, the I2 index does not tell us how much the effect size varies across studies. J Physiother.

[27] Egger M, Davey Smith G, Schneider M, Minder C (1997). Bias in meta-analysis detected by a simple, graphical test. BMJ.

[28] Begg CB, Mazumdar M (1994). Operating characteristics of a rank correlation test for publication bias. Biometrics.

[29] Wickham H. ggplot2: Elegant graphics for data analysis. New York: Springer-Verlag; 2016.

[30] Slowikowski K. ggrepel: automatically position non-overlapping text labels with ‘ggplot2’. R package version 0.9.1. 2021. https://CRAN.R-project.org/package=ggrepel

[31] Borgwardt SJ, Riecher-Rössler A, Dazzan P, Chitnis X, Aston J, Drewe M (2007). Regional gray matter volume abnormalities in the at risk mental state. Biol Psychiatry.

[32] van Tricht MJ, Nieman DH, Koelman JHTM, van der Meer JN, Bour LJ, de Haan L (2010). Reduced parietal P300 is associated with an increased risk for a first psychotic episode. Biol Psychiatry.

[33] Walker EF, Brennan PA, Esterberg M, Brasfield J, Pearce B, Compton MT (2010). Longitudinal changes in cortisol secretion and conversion to psychosis in at-risk youth. J Abnorm Psychol.

[34] Bearden CE, Wu KN, Caplan R, Cannon TD (2011). Thought disorder and communication deviance as predictors of outcome in youth at clinical high risk for psychosis. J Am Acad Child Adolesc Psychiatry.

[35] Liu CC, Lai MC, Liu CM, Chiu YN, Hsieh MH, Hwang TJ (2011). Follow-up of subjects with suspected prepsychotic state in Taiwan. Schizophr Res.

[36] Ziermans TB, Schothorst PF, Sprong M, van Engeland H (2011). Transition and remission in adolescents at ultra-high risk for psychosis. Schizophr Res.

[37] Schlosser DA, Jacobson S, Chen Q, Sugar CA, Niendam TA, Li G (2012). Recovery from an at-risk state: clinical and functional outcomes of putatively prodromal youth who do not develop psychosis. Schizophr Bull.

[38] Katsura M, Ohmuro N, Obara C, Kikuchi T, Ito F, Miyakoshi T (2014). A naturalistic longitudinal study of at-risk mental state with a 2.4 year follow-up at a specialized clinic setting in Japan. Schizophr Res.

[39] DeVylder JE, Muchomba FM, Gill KE, Ben-David S, Walder DJ, Malaspina D (2014). Symptom trajectories and psychosis onset in a clinical high-risk cohort: the relevance of subthreshold thought disorder. Schizophr Res.

[40] Perez VB, Woods SW, Roach BJ, Ford JM, McGlashan TH, Srihari VH (2014). Automatic auditory processing deficits in schizophrenia and clinical high-risk patients: forecasting psychosis risk with mismatch negativity. Biol Psychiatry.

[41] Schultze-Lutter F, Klösterkotter J (2014). Improving the clinical prediction of psychosis by combining ultra-high risk criteria and cognitive basic symptoms. Schizophr Res.

[42] Bedi G, Carrillo F, Cecchi G, Slezak DF, Sigman M, Mota NB (2015). Automated analysis of free speech predicts psychosis onset in high-risk youths. NPJ Schizophr.

[43] Katagiri N, Pantelis C, Nemoto T, Zalesky A, Hori M, Shimoji K (2015). A longitudinal study investigating sub-threshold symptoms and white matter changes in individuals with an ‘at risk mental state’ (ARMS). Schizophr Res.

[44] Labad J, Stojanovic-Perez A, Montalvo I, Solé M, Cabezas Á, Ortega L (2015). Stress biomarkers as predictors of transition to psychosis in at-risk mental states: roles for cortisol, prolactin and albumin. J Psychiatry Res.

[45] Brucato G, Masucci MD, Arndt LY, Ben-David S, Colibazzi T, Corcoran CM (2017). Baseline demographics, clinical features and predictors of conversion among 200 individuals in a longitudinal prospective psychosis-risk cohort. Psychol Med.

[46] Kotlicka-Antczak M, Pawełczyk A, Karbownik MS, Pawełczyk T, Strzelecki D, Żurner N (2017). Deficits in the identification of pleasant odors predict the transition of an at-risk mental state to psychosis. Schizophr Res.

[47] Collin G, Seidman LJ, Keshavan MS, Stone WS, Qi Z, Zhang T (2020). Functional connectome organization predicts conversion to psychosis in clinical high-risk youth from the SHARP program. Mol Psychiatry.

[48] Bang M, Park JY, Kim KR, Lee SY, Song YY, Kang JI (2019). Psychotic conversion of individuals at ultra-high risk for psychosis: the potential roles of schizotypy and basic symptoms. Early Inter Psychiatry.

[49] Hamilton HK, Woods SW, Roach BJ, Llerena K, McGlashan TH, Srihari VH (2019). Auditory and visual oddball stimulus processing deficits in schizophrenia and the psychosis risk syndrome: forecasting psychosis risk with P300. Schizophr Bull.

[50] Zarogianni E, Storkey AJ, Borgwardt S, Smieskova R, Studerus E, Riecher-Rössler A (2019). Individualized prediction of psychosis in subjects with an at-risk mental state. Schizophr Res.

[51] Bourgin J, Duchesnay E, Magaud E, Gaillard R, Kazes M, Krebs MO (2020). Predicting the individual risk of psychosis conversion in at-risk mental state (ARMS): a multivariate model reveals the influence of nonpsychotic prodromal symptoms. Eur Child Adolesc Psychiatry.

[52] Modinos G, Allen P, Zugman A, Dima D, Azis M, Samson C (2020). Neural circuitry of novelty salience processing in psychosis risk: association with clinical outcome. Schizophr Bull.

[53] Yoviene Sykes LA, Ferrara M, Addington J, Bearden CE, Cadenhead KS, Cannon TD (2020). Predictive validity of conversion from the clinical high risk syndrome to frank psychosis. Schizophr Res.

[54] Grent-‘t-Jong T, Gajwani R, Gross J, Gumley AI, Krishnadas R, Lawrie SM (2021). 40-Hz auditory steady-state responses characterize circuit dysfunctions and predict clinical outcomes in clinical high-risk for psychosis participants: a Magnetoencephalography Study. Biol Psychiatry.

[55] Nägele FL, Pasternak O, Bitzan LV, Mußmann M, Rauh J, Kubicki M (2021). Cellular and extracellular white matter alterations indicate conversion to psychosis among individuals at clinical high-risk for psychosis. World J Biol Psychiatry.

[56] Demars F, Kebir O, Marzo A, Iftimovici A, Schramm C (2020). ICAAR Study Group, et al. Dysregulation of peripheral expression of YWHA genes during conversion to psychosis. Sci Rep.

[57] Kristensen TD, Glenthøj LB, Ambrosen K, Syeda W, Ragahava JM, Krakauer K (2021). Global fractional anisotropy predicts transition to psychosis after 12 months in individuals at ultra-high risk for psychosis. Acta Psychiatr Scand.

[58] Tateno T, Higuchi Y, Nakajima S (2021). Features of duration mismatch negativity around the onset of overt psychotic disorders: a longitudinal study. Cereb Cortex.

[59] Yung AR, Yuen HP, McGorry PD, Phillips LJ, Kelly D, Dell’Olio M (2005). Mapping the onset of psychosis: the comprehensive assessment of at-risk mental states. Aust N Z J Psychiatry.

[60] Kay SR, Fiszbein A, Opler LA (1987). The positive and negative syndrome scale (PANSS) for schizophrenia. Schizophr Bull.

[61] Riecher-Rossler A, Aston J, Ventura J, Merlo M, Borgwardt S, Gschwandtner U (2008). The Basel Screening Instrument for Psychosis (BSIP): development, structure, reliability and validity. Fortschr Neurol Psychiatr.

[62] McGlashan TH. Structured Interview for Prodromal Symptoms (SIPS). Yale, CT, USA: Yale University; 2001.

[63] Li M (2016). Antipsychotic-induced sensitization and tolerance: Behavioral characteristics, developmental impacts, and neurobiological mechanisms. J Psychopharm.

[64] Chouinard G, Samaha AN, Chouinard VA, Peretti CS, Kanahara N, Takase M (2017). Antipsychotic-induced supersensitivity psychosis: pharmacological criteria and therapy. Psychother Psychosom.

[65] Nakata Y, Kanahara N, Iyo M (2017). Dopamine supersensitivity psychosis in schizophrenia: Concepts and implications in clinical practice. J Psychopharmacol.

[66] Yin J, Barr AM, Ramos-Miguel A, Procyshyn RM (2017). Antipsychotic induced dopamine supersensitivity psychosis: a comprehensive review. Curr Neuropharmacol.

[67] Chouinard G, Chouinard VA (2008). Atypical antipsychotics: CATIE study, drug-induced movement disorder and resulting iatrogenic psychiatric-like symptoms, supersensitivity rebound psychosis and withdrawal discontinuation syndromes. Psychother Psychosom.

[68] Kimura H, Kanahara N, Iyo M (2021). Rationale and neurobiological effects of treatment with antipsychotics in patients with chronic schizophrenia considering dopamine supersensitivity. Behav Brain Res..

[69] Lugg W (2022). Antipsychotic-induced supersensitivity -a reappraisal. Aust N Z J Psychiatry.

[70] McGorry PD, Yung AR, Phillips LJ, Yuen HP, Francey S, Cosgrave EM (2002). Randomized controlled trial of interventions designed to reduce the risk of progression to first-episode psychosis in a clinical sample with subthreshold symptoms. Arch Gen Psychiatry.

[71] McGlashan TH, Zipursky RB, Perkins D, Addington J, Miller T, Woods SW (2006). Randomized, double-blind trial of olanzapine versus placebo in patients prodromally symptomatic for psychosis. Am J Psychiatry.

[72] McGorry PD, Nelson B, Phillips LJ, Yuen HP, Francey SM, Thampi A (2013). Randomized controlled trial of interventions for young people at ultra-high risk of psychosis: twelvemonth outcome. J Clin Psychiatry.

