# Supplementary material for: The temporal dynamics of transition to psychosis in individuals at clinical high-risk (CHR-P) shows negative prognostic effects of baseline antipsychotic exposure: a meta-analysis
Source: Transl Psychiatry. 2023 Apr 5;13:112. doi: 10.1038/s41398-023-02405-6 (PMC10076303; doi:10.1038/s41398-023-02405-6)
Supplement: Supplementary file 1 — Supplementary materials [file 41398_2023_2405_MOESM1_ESM.docx]

**Figure S1.** Risk of bias summary plot according to the Newcastle-Ottawa Scale. Quality assessment of the included studies on CHR who were or were not exposed to antipsychotics at baseline.

**Figure S2**. Funnel plot (on the left) and the results of the Egger’s and the Begg’s test for the global meta-analysis of the risk ratio of conversion to psychosis between CHR who were or were not exposed to antipsychotics at baseline.

**Figure S3**. Forest plot of the risk ratio of conversion to psychosis between CHR who were or were not exposed to antipsychotics at baseline after exclusion of four studies with excess (>20%) dropouts.

**Figure S4**. Best fit of the cumulative transition curves (linear and quadratic) in CHR patients who were exposed (AP+) or not exposed (AP-) to antipsychotics at baseline
