# Supplementary material for: The temporal dynamics of transition to psychosis in individuals at clinical high-risk (CHR-P) shows negative prognostic effects of baseline antipsychotic exposure: a meta-analysis
Source: Transl Psychiatry. 2023 Apr 5;13:112. doi: 10.1038/s41398-023-02405-6 (PMC10076303; doi:10.1038/s41398-023-02405-6)
Supplement: Supplementary file 2 — Table S1 [file 41398_2023_2405_MOESM2_ESM.docx]

**Table S1** Quality rating of studies included in the Meta-analysis according to the Newcastle-Ottawa Assessment Form for Cohort Studies.

| **Study** | **Selection** | | | | **Comparability** | **Outcome** | | | **AHRQ**  **Standard** |
| --- | --- | --- | --- | --- | --- | --- | --- | --- | --- |
|  | **Item 1** | **Item 2** | **Item 3** | **Item 4** | **Item 1** | **Item 1** | **Item 2** | **Item 3** |  |
| Borgwardt et al. (2007) | * | * | * | * | * | * | * | * | Good |
| van Tricht et al. (2010) | * | * | * | * | * | * | * | * | Good |
| Walker et al., (2010) | * | * | * | * |  | * | * | * | Good |
| Bearden et al. (2011) | * | * | * | * | * | * | * | * | Good |
| Liu et al., (2011) | * | * | * | * | * | * | * | * | Good |
| Ziermans et al., (2011) | * | * | * | * |  | * | * | * | Good |
| Schossler et al., (2012) | * | * | * | * | * | * | * |  | Good |
| Katsura et al., (2014) | * | * | * | * |  | * | * |  | Poor |
| De Vylder et al., (2014) | * | * | * | * |  | * | * | * | Good |
| Perez et al., (2014) | * | * | * | * | * | * | * | * | Good |
| Schultze-Lutter et al., (2014) | * | * | * | * |  | * | * |  | Poor |
| Bedi et al., (2015) | * | * |  |  |  | * | * | * | Poor |
| Katagiri et al., (2015) | * | * | * | * | * | * | * | * | Good |
| Labad et al., (2015) | * | * | * | * | * | * | * | * | Good |
| Brucato et al., (2017) | * | * | * | * |  | * | * | * | Good |
| Kotlicka-Antczak et al. (2017) | * | * | * | * |  | * | * | * | Good |
| Collin et al., (2018) | * | * | * | * | * | * | * | * | Good |
| Bang et al., (2019) | * | * | * | * | * | * | * | * | Good |
| Hamilton et al., (2019) | * | * | * | * | * | * | * | * | Good |
| Zarogianni et al., (2019) | * | * | * | * |  | * | * | * | Good |
| Modinos et al., (2020) | * | * | * | * | * | * | * | * | Good |
| Yoviene-Sikes et al., (2020) | * | * | * | * |  | * | * |  | Poor |
| Demars et al., (2020) | * | * | * | * |  | * | * |  | Poor |
| Bourgin et al., (2020) | * | * | * | * |  | * | * |  | Poor |
| Nagele et al., (2020) | * | * | * | * | * | * | * | * | Good |
| Grent't-Jong et al., (2021) | * | * | * | * | * | * | * | * | Good |
| Kristensen et al., (2021) | * | * | * | * |  | * | * | * | Good |
| Tateno et al., (2021) | * | * | * | * | * | * | * | * | Good |

*Note: Newcastle-Ottawa Quality Assessment Form for Cohort Studies*

*Selection items*

*1) Representativeness of the exposed cohort*

*2) Selection of the non-exposed cohort*

*3) Ascertainment of exposure*

*4) Demonstration that outcome of interest was not present at start of study*

*Comparability Items*

*1) Comparability of cohorts on the basis of the design or analysis controlled for confounders*

*Outcome items*

*1) Assessment of outcome*

*2) Was follow-up long enough for outcomes to occur*

*3) Adequacy of follow-up of cohorts*

*Thresholds for converting the Newcastle-Ottawa scales to AHRQ standards (good, fair, and poor):*

*Good quality: 3 or 4 stars in selection domain AND 1 or 2 stars in comparability domain AND 2 or 3 stars in outcome/exposure domain*

*Fair quality: 2 stars in selection domain AND 1 or 2 stars in comparability domain AND 2 or 3 stars in outcome/exposure domain*

*Poor quality: 0 or 1 star in selection domain OR 0 stars in comparability domain OR 0 or 1 stars in outcome/exposure domain*
