# Supplementary figures and images for: The temporal dynamics of transition to psychosis in individuals at clinical high-risk (CHR-P) shows negative prognostic effects of baseline antipsychotic exposure: a meta-analysis
Source: Transl Psychiatry. 2023 Apr 5;13:112. doi: 10.1038/s41398-023-02405-6 (PMC10076303; doi:10.1038/s41398-023-02405-6)

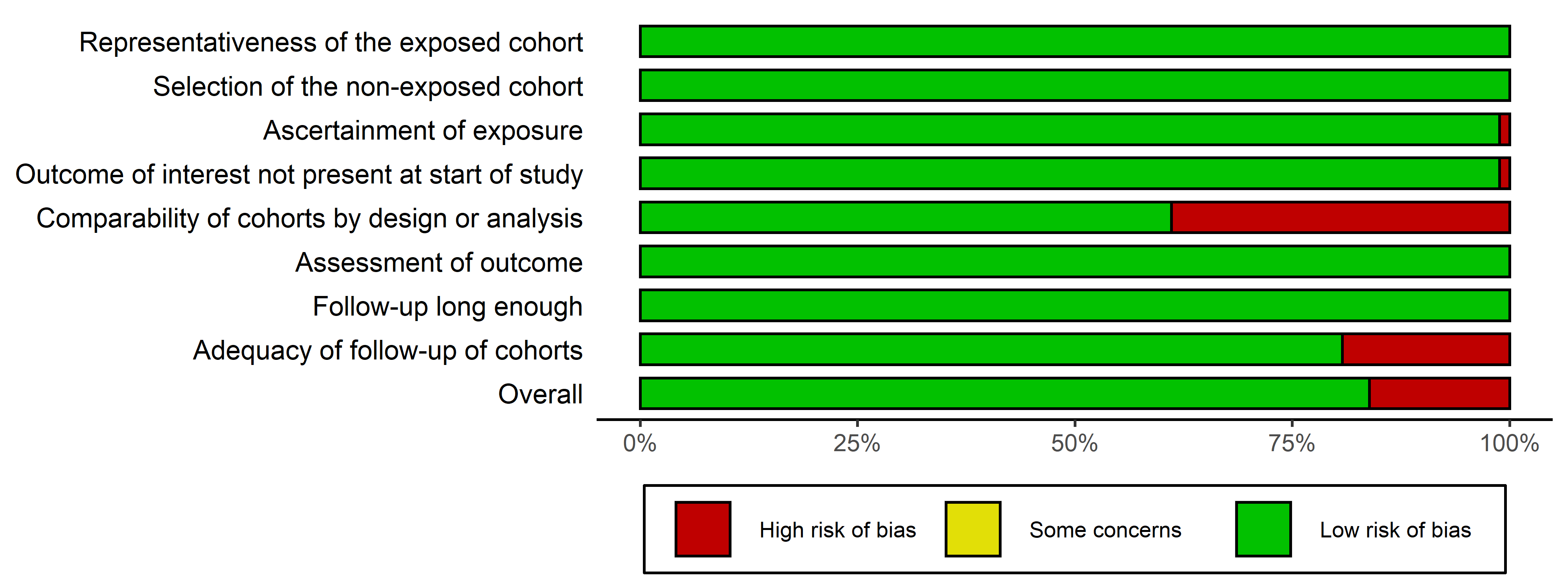

Supplement: Supplementary file 3 — Figure S1 [file 41398_2023_2405_MOESM3_ESM.tif]

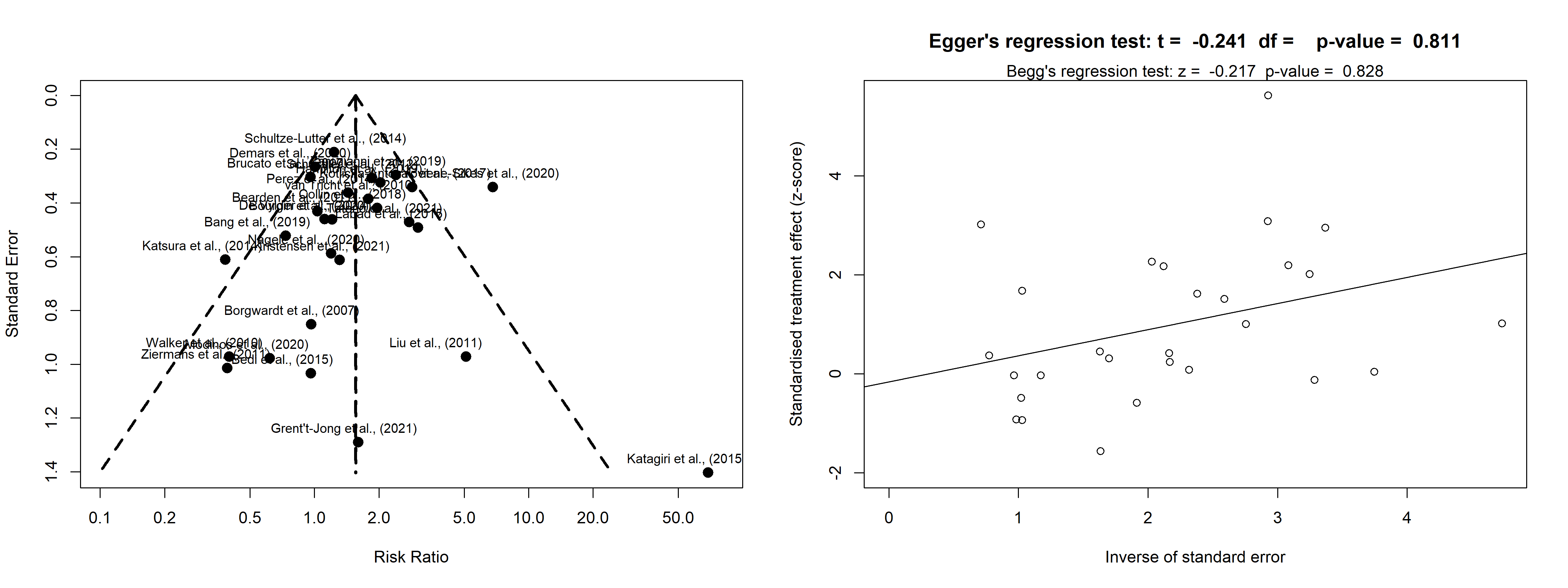

Supplement: Supplementary file 4 — Figure S2 [file 41398_2023_2405_MOESM4_ESM.tif]

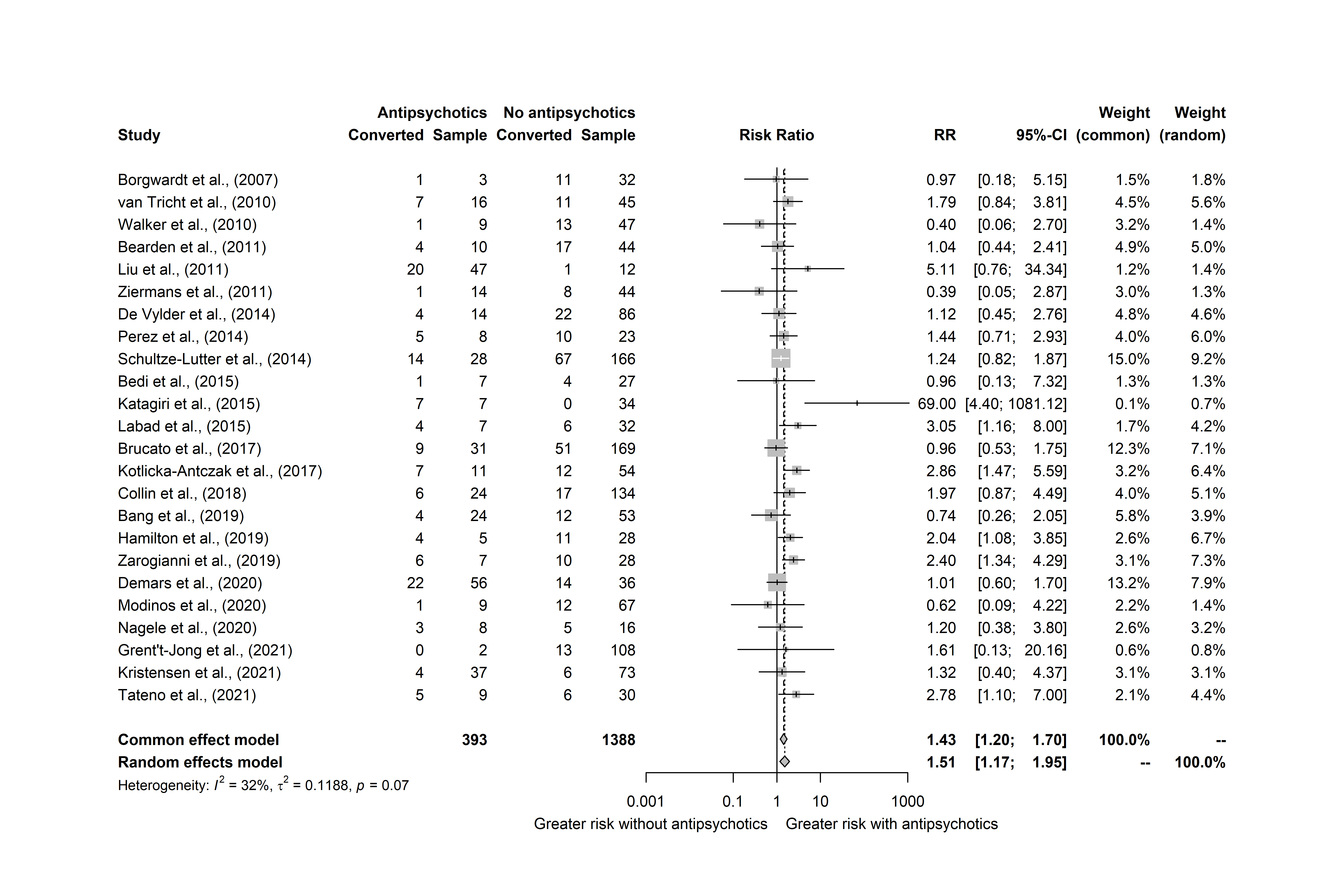

Supplement: Supplementary file 5 — Figure S3 [file 41398_2023_2405_MOESM5_ESM.tif]

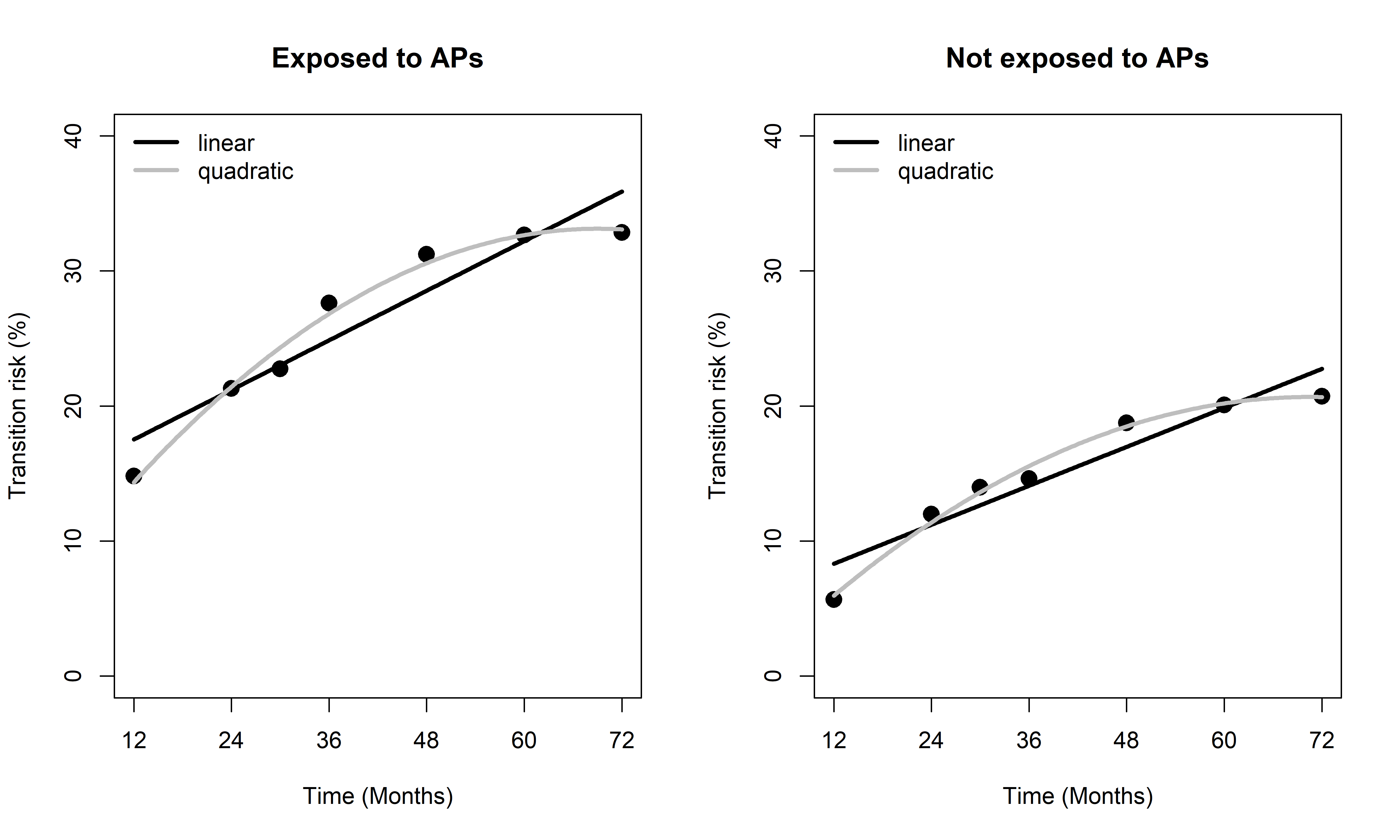

Supplement: Supplementary file 6 — Figure S4 [file 41398_2023_2405_MOESM6_ESM.tif]
